# Short-term effect of internet-delivered mindfulness-based stress reduction on mental health, self-efficacy, and body image among women with breast cancer during the COVID-19 pandemic

**DOI:** 10.3389/fpsyg.2022.949446

**Published:** 2022-10-25

**Authors:** Yun-Chen Chang, Chang-Fang Chiu, Chih-Kai Wang, Chen-Teng Wu, Liang-Chih Liu, Yao-Chung Wu

**Affiliations:** ^1^School of Nursing and Graduate Institute of Nursing, China Medical University, Taichung, Taiwan; ^2^Nursing Department, China Medical University Hospital, Taichung, Taiwan; ^3^Division of Hematology and Oncology, Department of Internal Medicine, China Medical University Hospital, China Medical University, Taichung, Taiwan; ^4^Cancer Center, China Medical University Hospital, Taichung, Taiwan; ^5^Department of Surgery, China Medical University Hospital, Taichung, Taiwan; ^6^Division of Breast Surgery, China Medical University Hospital, Taichung, Taiwan; ^7^School of Medicine, China Medical University, Taichung, Taiwan

**Keywords:** internet-delivered therapy, internet-delivered MBSR, breast cancer, mental health, self-efficacy, body image

## Abstract

**Background and aim:**

During the COVID-19 pandemic, an Internet-Mindfulness-Based Stress Reduction (iMBSR) program was delivered and may be better than an in-person approach. Our study evaluated the effects of iMBSR intervention on mental health, self-efficacy, and body image in women with breast cancer in Taiwan.

**Materials and methods:**

Sixty-seven women with breast cancer were allocated to a 6-week iMBSR (*n* = 41) program or a waitlist control group (*n* = 26), without heterogeneity between group characteristics. Patients from both groups were measured at baseline and postintervention using three scales: Depression, Anxiety, and Stress Scale (DASS-21), General self-efficacy scale, and Body Image Scale. Descriptive dataset analysis, paired *t*-test, and Student’s *t*-test were used to evaluate the data.

**Results:**

Although iMBSR did not significantly improve depression and stress between groups, iMBSR could improve anxiety (Δmean: −2.0 vs. −0.4, *p* = 0.041) with medium effect sizes. Significant benefits were found for body image (Δmean: −3.6 vs. 0.9, *p* = 0.003) and self-efficacy (Δmean: 4.2 vs. 1.5, *p* = 0.004), with large effect sizes (Cohen’s *d* = 0.73).

**Conclusion:**

Our preliminary study supports iMBSR as a program that can improve mental health, body image, and self-efficacy in women with breast cancer. During the COVID-19 pandemic, medical professionals can use Internet-based clinical health education.

## Introduction

A 2019 cancer registry report indicated that breast cancer had the second highest mortality rate in Taiwan and the highest incidence among cancers in women and increased by ~4.49% per year (Taiwan, Ministry of Health and Welfare, 2019).

Adverse effects of breast cancer treatment, such as cognitive impairments (Brown et al., 2021), alopecia and body scarring, and the removal of the breast, ovaries, or uterus, negatively affect gender role socialization and body image disturbance (Boquiren et al., 2013, 2016). Body image disturbance has been linked to depression, anxiety (Szymanski and Henning, 2007), sexual function (Chang et al., 2019), self-esteem, and quality of life (Richard et al., 2019). Consequently, interventions focus on reducing female body dissatisfaction are a critical topic of research (Wade et al., 2009).

Based on Bandura’s theory, self-efficacy refers to the ability of an individual to control their motivation, behavior, and how they face obstacles. The theory essentially attributes an individual’s belief in efficacy to their chance of achieving success in a particular situation (Bandura et al., 1999). Therefore, coping self-efficacy is a key determinant of a person’s ability to successfully manage stressful situations and emotions (Chesney et al., 2006). Low coping self-efficacy is associated with stress, depression, anxiety, and feelings of helplessness, whereas higher levels promote active and effective engagement in regulating emotional distress (Zulkosky, 2009). Self-efficacy is the belief that individuals can demonstrate positive attitudes toward healthy behaviors and directly affect their health-promoting behaviors (Stuifbergen et al., 2000).

Studies have highlighted that mindfulness-based interventions have a wide range of benefits, including decreased perceived stress, anxiety, depression, and fatigue (Price-Blackshear et al., 2020), improvements in sexual functioning (Bober et al., 2020), and increased self-efficacy in stress perception (Caldwell et al., 2010). Mindfulness interventions derived from ancient Buddhist and Hatha Yoga traditions are becoming increasingly popular in the Western world. The “way of being,” or to be aware in each moment, is the core of mindfulness and is achieved by “paying attention in a particular way: on purpose, in the present moment, and nonjudgmentally” (Kabat-Zinn, 1990). Over the past decade, mindfulness-based stress reduction (MBSR) interventions have grown rapidly in oncology research (Chang et al., 2021).

With the outbreak of COVID-19 in December 2019 (Wang et al., 2020a) and its rapid spread worldwide, public fear and heightened psychological symptoms have become common (Brooks et al., 2020; Wang et al., 2020b). The pandemic has increased the use of information and communication technology (ICT) interventions to help patients with breast cancer connect (Compen et al., 2019). ICT interventions can help patients comply with necessary epidemic prevention regulations while reducing the distance, time, and cost issues that arise when building and maintaining social connections and exchanging social support, thereby protecting the health of vulnerable populations (McCue et al., 2010; Galiano-Castillo et al., 2016; Compen et al., 2018).

Patients with breast cancer are at higher risk of psychological stress, and the additional stress of the COVID-19 pandemic can increase their vulnerability (Kang et al., 2021). Internet-delivered MBSR (iMBSR) improved psychological health, sleep quality, and life satisfaction in college students and young working adults (Mak et al., 2017). Goetz et al. (2020) found that applying electronic-mindfulness-based interventions (eMBI) can alleviate depression and anxiety symptoms in pregnant women at high risk of hospitalization (Goetz et al., 2020). Nourian et al. (2021) conducted an online MBSR program that improved sleep disturbance among nurses in a COVID-19 intensive care unit (Nourian et al., 2021).

A study in Denmark involving women with breast cancer and older men with prostate cancer who underwent an Internet-delivered MBI (iMBI) program found that iMBIs could improve anxiety and depressive symptoms immediately after the intervention (Nissen et al., 2020). Another study compared the effects of iMBCT with face-to-face mindfulness-based cognitive therapy (MBCT) in patients with cancer, and concluded that both interventions reduced psychological distress (Compen et al., 2018).

Considerable research explores the effects of iMBSR on physical and psychological problems in patients without cancer, and using iMBCT to resolve emotional distress in patients with cancer. However, there has been little research on iMBSR’s effects on self-efficacy and body image in women with breast cancer. Therefore, our study aimed to explore the effect and feasibility of iMBSR on mental health, self-efficacy, and body image in women with breast cancer during COVID-19.

## Materials and methods

### Participant inclusion and exclusion criteria

Both the iMBSR and waitlist control group used purposive and snowball sampling. Potential participants were recruited from visiting breast surgery clinics, cancer wards, and breast cancer affiliate websites. Inclusion criteria were as follows: had been diagnosed with stage 0–IV breast cancer within the past 5 years; aged ≥20 years old; could communicate in Mandarin; received at least recent adjuvant therapy (i.e., chemotherapy, radiotherapy, or hormonal therapy); and Eastern Cooperative Oncology Group performance score ≤ 1 (Taylor et al., 1999). Exclusion criteria were as follows: a history of psychiatric diagnosis (e.g., acute psychosis) and suicide tendencies.

### Procedure

Our study has several required online software to facilitate online teaching and recruiting participants (Table 1). The recruitment period was from 1st February to 1st March, 2022. The study coincided with the official declaration of the COVID-19 outbreak by the Taiwan Centers for Disease Control on 9 March, 2022, which prohibited close activities with others (Taiwan Centers for Disease Control (TCDC), 2022). Patients with cancer receiving chemotherapy can have the adverse effect of neutropenia which can increase their risk of serious infection (Crawford et al., 2004); therefore, all data would be collected online through Google Forms.

**Table 1:** Web-based collaboration (or interactive) software (or APP).

Online software	Purpose of usage
Google	
Google Forms	Recruiting Participants Online form Pre-test questionnaire form Post-test questionnaire form Records of personal practice frequency and dosage, practice impressions, and awareness experience
Jamboard	Sign-in form for check participation rate
Microsoft Teams	A communication platform used as a teaching tool for online mindfulness courses
LINE App	Share our Microsoft Teams meet link Communication tool for when participants could not access Microsoft Teams Reminder of course time A medium for verbal encouragement and used to enhance self-efficacy Share personal experience of daily practice
7-ELEVEN App	E-coupons can be stored

#### Data collection

All participants submitted written informed consent during the orientation. Variable measurement was performed at two time points, including pretest and posttest. (1) Pretest: This was completed before the program’s second session. Baseline scores were based on the participants; responses to items across the scale. (2) Posttest: After the sixth week of training, participants completed a posttest within a week. Participants received a TWD 100 electronic coupon after completing the pre and postintervention questionnaires.

##### Internet-based MBSR intervention

The frequency and duration of our mindfulness training were six 2-h weekly group coaching sessions led by a professionally trained psychologist using online learning with Microsoft Teams. We used Jamboard, a digital interactive whiteboard developed by Google, to measure the number of patients participating online before class. Our training aimed to improve these metrics: mindful eating, body scan, breath awareness practice, mindful walking, and sharing of group experiences. The informal training in our study was conducted through home practice using mindfulness-guided cloud-based applications such as YouTube (Chang et al., 2022). Specifically, participants performed 10–15 min of home practice at least twice daily, listened for at least half an hour before sleep, and recorded their personal experiences on a Google Forms survey. Informal practice does not require a specific amount of time and can be practiced anytime and anywhere. The informal training aimed to improve overall self-awareness, communication, learning, and listening. Although formal training is more widely discussed and practiced, supplemental informal training may be more helpful for participants to adopt mindful attitudes in their lives (Table 2).

**Table 2:** The content of iMBSR.

Sessions	Contents	Mindfulness practice	Homework
1	Introduction to the class, how to proceed, requirements and challenges Introduce the relationship between brain function, emotion, and cognition Introduce the “fight or flight, freeze” stress response Internal and external interactions: the relationship between situation (stimuli), thoughts, sensations, bodily sensations, and actions Differences between “past,” “future,” and present Awareness and “Autopilot Mode”	Motivation and Intention: Why am I here? Identify concerns Mindful Eating	Mindful Eating Self-awareness of worry (or other strong emotions)
2	The negative cycle of worry How thoughts affect mood, physical feelings ntroduce the cognitive model (stimuli-intrinsic responses [thoughts, sensations, bodily sensations]-actions) The mode of doing and the mode of being of the mind “Myocardial training”: focus and awareness	Mindful Eating Breath awareness practice Be aware of the negative cycle of worry	The negative cycle of worry Breath awareness practice Mindful Eating
3	See the chaotic mind, from the breath into the presence mode Be aware of the distraction and gently bring the focus back to the breath Be aware of the inertial reaction pattern, and the emotional cycle is wave after wave Resist unwanted, disliked, unpleasant Ideas are not facts The Second Arrow Theory of Suffering (Pain and Suffering)	Breath awareness practice (Long Version) Awareness of inertial response patterns Distinguish pain and suffering	Breath awareness exercises when strong emotions arise Awareness of inertia and shooting arrows at yourself (automatic thinking) Mindfulness in daily life (showering, brushing teeth…)
4	Experience the true meaning of acceptance Story: Fear in the Heart – There are tigers in the closet From rejection to acceptance, not into action mode Take care of yourself Introduce the principles of mindfulness	Breath awareness practice (Long Version) Three minutes breathing room Mindful walking	Stress awareness Take 3 min of breathing space during strong emotions Mindfulness in daily life (breathing into life)
5	Introducing S.T.O.P. Application of Breath Awareness Mindfulness guards the mood, not following or resisting Observe the changes of mind and body and control inexplicable emotions Difficulties encountered in mindfulness practice	Three minutes breathing room Body scan	S.T.O.P. – Stress Reaction and Response Body scan Daily practice: focus on talking to people A letter to myself (reflection lessons and outlook for future mindfulness activities)
6	Common reactions to body scans To be aware of the relationship between change and discomfort Explore with an open mind Thoughts are not me, feelings are not me, just a part of me Mindful listening The concept of loving-kindness	Three minutes breathing room Metta meditation practice Review the difficulties of the course and bless yourself	Mindfulness practice for living

#### Waitlist control group

Inclusion and exclusion criteria for WCG (delay in receiving the intervention) were the same as for the iMBSR group. WCG occurred in parallel with iMBSR. The WCG had manuals for managing symptoms and oral instructions on health and hygiene education. All participants were instructed not to participate in any other stress reduction program or mind–body therapy during the study. We offered an additional iMBSR skills study program to WCG participants following our study’s conclusion.

#### Ethics consideration

The present study involved research with human participants who were required to sign an informed consent form before participating in the study. All procedures in our research comply with the ethical standards of the Institutional Research Council.

### Mental health, self-efficacy, and body image measures

#### Main research tools

##### Depression, anxiety, and stress

The Depression, Anxiety, and Stress Scale (DASS-21) is a 21-item tool that evaluates the participant’s depression, anxiety, and stress over the past week, on a Likert-style scale ranging from 0 (never) to 3 (almost always; Lovibond and Lovibond, 1995). Higher scores indicate greater anxiety, depression, or stress index and vice versa (Lovibond and Lovibond, 1995). The DASS-Depression Inventory was correlated with the Baker Depression Inventory at 0.74 and the Anxiety Inventory with the Baker Anxiety Inventory at 0.81 (Lovibond and Lovibond, 1995). Wei et al. (2008) translated the DASS-21 scale into Chinese (Wei et al., 2008), and its psychometric properties were evaluated using an Australian immigrant sample (*n* = 356) and compared with Lovibond and Lovibond’s (1995) English version of the DASS (*n* = 720). Multi-group confirmatory factor analysis found that the Chinese version of the DASS-21 effectively discriminates between depression, anxiety, and stress, but less so than the English version.

##### The body image scale

In collaboration with the European Organization for Research and Treatment of Cancer Quality of Life Study Group, Hopwood et al. constructed a 10-item Body-Mental Imagery Scale using datasets from seven treatment trials/clinical studies in the United Kingdom on 682 patients with breast cancer. Psychological testing was conducted and the scale showed high reliability (Cronbach’s alpha 0.93) and good discriminant validity (*p* < 0.0001), with sensitivity to change (*p* < 0.001; Hopwood et al., 2001). Scores are calculated by totaling items on a scale of 0 (not at all) to 3 (very), with higher scores indicating greater body image impairment and vice versa (Hopwood et al., 2001).

##### General self-efficacy scale

The General Self-Efficacy Scale (GSES), developed by Jerusalem and Schwarzer, is a generic tool that is widely used to measure self-efficacy in clinical and nonclinical populations (Zhang and Schwarzer, 1995; Zhang et al., 2018). Zhang et al. (2018) translated the GSES into Chinese, which consisted of 10 items, and used a 4-point Likert scale from 1 (always false) to 4 (always true). The Chinese version of the GSES (C-GSES) exhibited good internal consistency with a Cronbach’s alpha of 0.926 in a unidimensional factorial model (Zhang et al., 2018).

##### Basic demographic questionnaire

The questionnaire was divided into two parts. First: basic personal information, including age, marital status, and economic status. Second: disease status, including time since diagnosis (years), cancer staging, and cancer treatment. The questionnaire was collected using an online Google form.

#### Fidelity

To maintain the fidelity of our online mindfulness program, the 6-week intervention was conducted by a qualified clinical psychologist with specialized training in psychology at the Medical Center, Departmental Cancer Center. The instructor assessed the transfer of iMBSR skills by asking questions and discussing the material with the participants, and the patients recorded their practice duration weekly. The first author (YCC) confirmed that the practice had been completed during the intervention. We also prepared a standardized process. First, we set up a LINE messenger group with a mobile app before iMBSR to remind participants of the class schedule, provide class information, and solve the participants ‘problems during the course (such as being unable to login to Microsoft Teams, unable to turn on the speaker, and unable to hear sound). Second, we regularly provided support and addressed participants’ needs wherever possible. Third, we used the patient groups to care for each other and share experiences (as part of the curriculum, the grouping feature of Microsoft Teams divided participants into groups for easier discussion and teamwork). Finally, if the participants completed the program, we provided an e-coupon as a reward.

#### Sample size

The sample size of our study was calculated based on the results of our previous study (Chang et al., 2022). We assumed that the DASS-21-anxiety subscale score would decrease from 28.54 to 17.62, in the MBSR group, whereas the score would remain unchanged in the WCG. These estimates were in line with the DASS-21 minimum clinically significant differences. With 80% power and a 5% type I error to detect a medium effect size of 0.5, the required sample size was calculated to be 40 participants. We considered a potential dropout rate of ~20%, hence we selected 48 participants.

### Statistical analysis

Data analysis was performed using SPSS for Windows 22.0 (IBM, Chicago, IL, United States). The collected questionnaires were coded, and the accuracy of the data was verified repeatedly after input. Descriptive statistics were described for the baseline demographic and clinical characteristics of the patients with breast cancer. The Kolmogorov–Smirnov test (KS test) tested raw data for adherence to the assumptions of normality and equal variance and the detection of outliers before inferential statistical procedures. Self-report data between baseline and 6 weeks were compared for within-group and between-group data analyses using paired *t*-tests and Student’s *t*-tests for normally distributed continuous variables, respectively. The statistical significance level was set to a two-sided *p* < 0.05 in all statistical analyses.

To explore the magnitudes of difference between groups, Cohen suggested that effect sizes be categorized into small, medium, and large treatment effects and represented by 0.2, 0.5, and 0.8, respectively (Cohen, 1988). A website was established following Lenhard and Lenhard’s (2016) principle, which could be used to determine the effect size of the *t*-test results.^1^

## Results

### Patient characteristics

**Figure 1:**
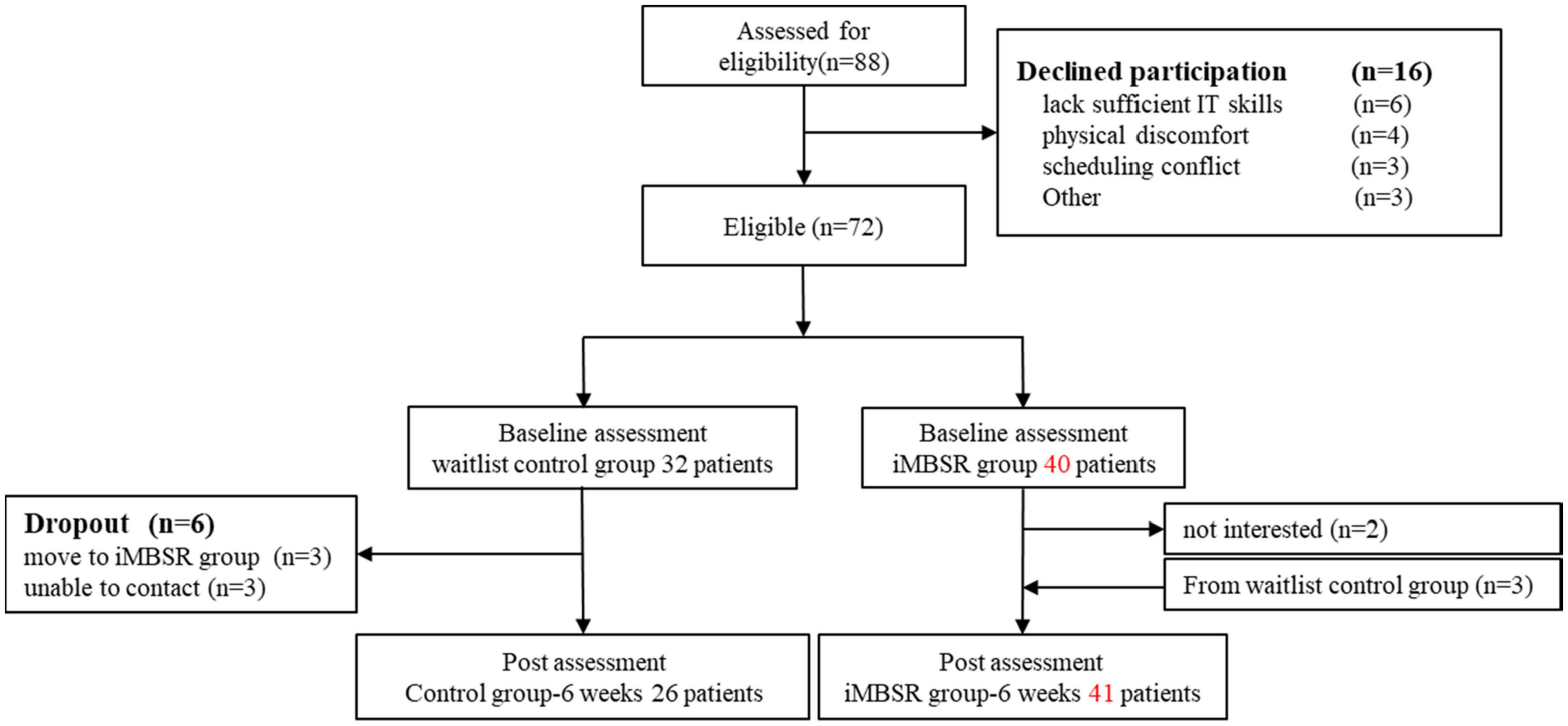
Flow diagram of study participants.

Our study had 88 eligible participants (see Figure 1 Flow diagram of study participants). The reasons for declining participation (*n* = 15) were lack of IT skills, physical discomfort, scheduling conflict, and others. We had established baseline data for 72 participants before the first session, with six dropouts in the WCG and three who changed to the Internet format. The postintervention assessment was completed by 41 and 26 participants in the MBSR and WCGs, respectively. The demographic and clinical characteristics of the patients are listed in Table 3. All participants were women and their mean age was 49.61 ± 12.03. The average breast cancer diagnosis time was ≤2 years (55.2%), and 37 patients were diagnosed with stage II cancer (55.2%). Most participants were married (46, 68.7%), and 20 were Unmarried (29.9%). The average monthly income was NTD 10,001–70,000 (62.7%), with 11 participants earning ≥ NTD 70,001 (16.4%). During the study period, the most common treatment was hormone therapy (32, 47.8%). We found no significant differences in basic characteristic data between the experimental and WCGs, except for age (*p* = 0.043).

**Table 3:** The demographic and clinical characteristic of patients with breast cancer.

Characteristic	Total (*n* = 67)^a^	iMBSR (*n* = 41)^a^	WCG (*n* = 26)^a^	Value of *p*
Age, years (SD)	49.61 (12.03)	53.38 (13.04)	47.21 (10.83)	0.043
21–30	2 (3.0)	1 (2.4)	1 (3.8)	
31–40	17 (25.4)	14 (34.1)	3(11.5)	
41–50	25 (37.3)	15 (36.6)	10 (38.5)	
51–60	21 (31.3)	11 (26.8)	10 (38.5)	
≥61	2 (3.0)	0 (0.0)	2 (7.7)	
Time since diagnosis, years, *n* (%)				0.314
≤2	37 (55.2)	25 (61.0)	12 (46.1)	
3–5	23 (34.4)	13 (31.7)	10 (38.4)	
≥5	7 (10.4)	3 (7.3)	4 (15.4)	
Marital status, *n* (%)				0.210
Unmarried	20 (29.9)	9 (22.0)	11 (42.3)	
Married	46 (68.7)	32 (78.0)	14 (53.8)	
Divorced	1 (1.5)	0 (0.0)	1 (3.8)	
Average monthly income in TWD, *n* (%)				0.445
≤10,000	10 (14.9)	7 (17.1)	3 (11.5)	
10,001–70,000	42 (62.7)	26 (63.4)	16 (61.5)	
≥70,001	11 (16.4)	7 (17.1)	4 (15.4)	
House keeper	4 (6.0)	1 (2.4)	3 (11.5)	
Cancer staging, *n* (%)				0.102
Stage Ο	4 (6.0)	3 (7.3)	1 (3.8)	
Stage I	13 (19.4)	10 (24.4)	3 (11.5)	
Stage II	37 (55.2)	21 (51.2)	16 (61.5)	
Stage III	6 (9.0)	5 (12.2)	1 (3.8)	
Stage IV	7 (10.4)	2 (4.9)	5 (19.2)	
Treatment, *n* (%)				0.924
Chemotherapy	26 (38.8)	16 (39.0)	10 (38.5)	
Radiotherapy	3 (4.5)	2 (4.9)	1 (3.8)	
Hormone therapy	32 (47.8)	20 (48.8)	12 (46.2)	
Other	6 (9.0)	3 (7.3)	3 (11.5)	
Single or both breasts, *n* (%)				0.955
Single	62 (92.5)	38 (92.7)	24 (92.3)	
Both	5 (7.5)	3 (7.3)	2 (7.7)	

a Categorical variable were presented as frequencies and percentages, continuous variables were presented as mean and standard deviation. iMBSR, Internet-Based Mindfulness-Based Stress Reduction; WCG, Waitlist Control Group; SD, Standard Deviation.

### Effects of internet-delivered MBSR

We used DASS-21 to measure participants’ current psychological problems. The DASS-21 results indicated significantly improved anxiety (Δmean: −2.0 vs. −0.4, *p* = 0.041). However, depression (Δmean: −1.5 vs. −1.5, *p* = 0.918), stress (Δmean: −2.1 vs. 0.5, *p* = 0.277), and overall DASS-21 scores (Δmean: −5.56 vs. −1.38, *p* = 0.243) were not significantly decreased. Six consecutive weeks of iMBSR sessions also improved body image (Δmean: −3.6 vs. 0.9, *p* = 0.003) and self-efficacy (Δmean: 4.2 vs. 1.5, *p* = 0.004) in women with breast cancer.

Additionally, the effect size of the two groups after the intervention differed in each outcome. The negative body image had large effect sizes, anxiety had medium effect sizes, and stress had small effect sizes; however, effect sizes < *d* = 0.2 were considered as having no treatment effect for overall DASS-21 and depression scores.

Our results indicated that those who had iMBSR experienced postintervention improvements to scores in overall DASS-21 (*p* = 0.019, 95% confidence interval [CI] = 0.27, 2.76) and significant decreases in the subscale of depression (*p* = 0.002, 95% CI = 0.79, 3.21), anxiety (*p* = 0.007, 95% CI = 0.60, 3.50), and stress (*p* = 0.001, 95% CI = 2.40, 8.72). The intervention significantly improved the participants’ negative body image (*p* = 0.000, 95% CI = 1.79, 5.43) and increased self-efficacy (*p* = 0.009, 95% CI = −7.22, −1.08; Table 4).

**Table 4:** The effect of pre and postintervention.

Characteristic	Group	Preintervention		Postintervention		Effect of intervention				
		Mean	SD	Mean	SD	ΔMean	*t*	Value of *p*^a^ (95% CI)	Value of *p*^b^ (95% CI)	Effect size^c^
DASS-21	WCG	37.73	15.13	36.35	6.71	-1.38	1.18	0.243 (−2.22, 8.62)	0.132 (−0.49, 3.49)	0.07
	iMBSR	38.71	13.17	33.15	12.74	−5.56			0.019^*^ (0.27, 2.76)	
Depression	WCG	12.19	5.66	10.69	2.13	−1.5	0.090	0.918 (−1.74, 1.91)	0.714 (−1.58, 2.28)	0.02
	iMBSR	12.12	4.28	10.61	4.33	−1.5			0.002^*^ (0.79, 3.21)	
Anxiety	WCG	12.23	5.13	11.88	2.63	−0.4	2.283	0.041^*^ (0.76, 3.65)	0.620 (−2.35, 1.43)	0.55
	iMBSR	12.02	4.64	10.02	4.04	−2.0			0.007^*^ (0.60, 3.50)	
Stress	WCG	13.31	5.77	13.77	3.66	0.5	0.997	0.277 (−1.03, 3.55)	0.481 (−3.43, 1.66)	0.26
	iMBSR	14.56	5.46	12.51	5.72	−2.1			0.001^**^ (2.40, 8.72)	
BIS	WCG	11.04	5.88	11.92	3.49	0.9	2.765	0.003^*^ (1.22, 5.75)	0.298 (−4.52, 1.45)	0.73
	iMBSR	12.05	6.98	8.44	5.78	−3.6			0.000^**^ (1.79, 5.43)	
GSES	WCG	10.58	5.70	12.12	4.35	1.5	−2.762	0.004^*^ (−6.77, −1.39)	0.579 (−3.69, 6.46)	0.73
	iMBSR	12.05	6.98	16.20	6.68	4.2			0.009^*^ (−7.22, −1.08)	

^*^p ≦ 0.05; ^**^p ≦ 0.001; Δpost-test minus pre-test scores; *t* Student *t*-test; a and c The score between group; b The score within group; DASS-21 Depression Anxiety Stress Scales-21; BIS The Body Image Scale; GSES General self-efficacy scale.

## Discussion

Few studies have been conducted on related topics, and this study complements the available research on the effects of Internet-delivered iMBSR to improve depression, anxiety, stress, body image, and self-efficacy in women with breast cancer.

The COVID-19 pandemic has increased the uptake and number of Internet services and electronic products, which has helped health professionals to provide online mental health education (Liu et al., 2020). Wei et al. (2020) applied comprehensive Internet-based interventions designed to improve relaxation, self-care, and feelings of individual safety and found that the symptoms of depression and anxiety in the intervention group were significantly lower than those in the control group (Wei et al., 2020). Some studies have found evidence of Internet-based mental health intervention program benefits, particularly for depression, anxiety, and stress, which is consistent with our findings. A large sample study (*N* = 1,282) found improvements in anxiety and depression in women with breast cancer following Internet-based Mindfulness-Based Cognitive Therapy (iMBCT; Nissen et al., 2020). However, in our study, we found no significant decrease in DASS-21 total scores, depression, and stress between the two groups. Possible reasons may be the small sample size of each group and the need for a longer intervention time to assess long-term effects.

Women treated for breast cancer experience many bodily changes, including the absence or deformity of one or both breasts, hair loss from chemotherapy, skin discolorations, and weight gain or loss. In the first few months after diagnosis, a large proportion of patients with breast cancer experienced changes in body image. Approximately 74.8% of women treated for breast cancer were dissatisfied with their body image (Guedes et al., 2018). Body image is seen as a highly subjective mental representation that reflects not only one’s physical appearance, body, and attractiveness but also one’s perceptions of mental health, marital quality, psychological stress, and perceived functioning (Boquiren et al., 2016; Farnam et al., 2021). Research has indicated that some patients with breast cancer experience one or ≥ two negative body image problems, accounting for 17% and 33%, respectively (Fobair et al., 2006). Therefore, it is significant that our results indicated that iMBSR improved body image and alleviated psychological problems. A potential reason why Internet-based MBSR improved body image was that women with breast cancer could avoid being stared at by others. Additionally, the Internet-based teaching method allowed the participants to practice mindfulness skills in a more comfortable place and in a relaxed manner.

Self-efficacy levels are essential in healthy behavioral change, and increases or decreases in self-efficacy can affect a person’s motivation to engage in actions (Redding et al., 2000). In our study, the mindfulness-based intervention appeared to be an effective approach to developing self-efficacy and helped participants increase their training in mindful healthy behaviors, encouraging them to generate positive thoughts and motivational changes on a task-specific basis. Individuals with breast cancer who have high levels of self-efficacy in mindfulness and medical management programs may more easily deal with complex situations such as building self-confidence, self-esteem, changes in body image, and appropriate medication management. High levels of self-efficacy may also lead to higher levels of motivation because they believe they can self-manage the conditions induced by breast cancer treatment (Zulkosky, 2009). A single-blind design study recruited 81 women facing high levels of physical and psychological stress and allocated them to either a waiting list (delayed start) control group or a meditation intervention group (Goldstein et al., 2018). These findings have critical implications for developing self-efficacy in coping with cancer and alleviating life stress in these vulnerable women (Goldstein et al., 2018). These studies indicated that MBSR could improve women’s self-efficacy, which was consistent with the findings of this study.

The COVID-19 pandemic meant that our intervention was Internet-based to better ensure that the study did not place women with breast cancer (and potentially with leukopenia) at risk of coronavirus infection (Kimura et al., 2021), thereby encouraging them to continue participating in the iMBSR intervention of distance learning. However, the 6-week intervention period may have been too brief to produce improvements in all areas. The most significant source of self-efficacy is constant practice because it relies on actual personal experience. Successful experiences help increase self-efficacy. Therefore, future studies can investigate the long-term effect of mindfulness skill practice on self-efficacy outcomes in breast cancer survivors. Larger samples would also strengthen the results.

### Strengths and limitations

Our study has several strengths. Our investigation was during the COVID-19 pandemic, highlighting the importance of an Internet-based psychoeducational intervention. The e-delivery prevented exhaustion from long commutes and reduced infection rates, which was critical for patients with cancer and potentially compromised immune systems. Additionally, we initially expected to enroll 48 participants, but we completed the final program with 67, exceeding our expected inclusion rate. However, this study had some limitations. First, we did not measure the long-term effects of iMBSR on patients with breast cancer. Second, the average age of the participant was 49.61 ± 12.03. Some older participants struggled with the Microsoft Teams software and could not join the course, hear the sound, or operate the digital whiteboard. Therefore, the staff should have spent more time contacting individuals struggling with the software.

## Conclusion

Overall, our findings indicate that we provided women with breast cancer with a convenient and cost-effective method to enhance their empowerment and confidence through mindfulness-based mental health education that taught them how to easily incorporate mindfulness techniques into their daily lives. We propose a future study investigating the acceptability and cost-effectiveness of Internet-based mindfulness interventions for people with different sociodemographic conditions.

## Data availability statement

The original contributions presented in the study are included in the article/supplementary material; further inquiries can be directed to the corresponding authors.

## Ethics statement

The studies involving human participants were reviewed and approved by Research Ethics Committee, China Medical University & Hospital, Taichung, Taiwan. The patients/participants provided their written informed consent to participate in present study.

## Author contributions

Y-CC: study conception and design. Y-CC, C-FC, C-KW, C-TW, L-CL, and Y-CW: data collection and critical revision of the article. Y-CC and C-KW: data analysis and interpretation and drafting of the article. All authors contributed to the article and approved the submitted version.

## Funding

This research was partially supported by a grant from China Medical University, Taiwan (CMU110-N-11 and CMU111-MF-115) and Ministry of Science and Technology, Taiwan (MOST 110-2314-B-039-059 and MOST 111-2314-B-039-016), received by Y-CC.

## Conflict of interest

The authors declare that the research was conducted in the absence of any commercial or financial relationships that could be construed as a potential conflict of interest.

## Publisher’s note

All claims expressed in this article are solely those of the authors and do not necessarily represent those of their affiliated organizations, or those of the publisher, the editors and the reviewers. Any product that may be evaluated in this article, or claim that may be made by its manufacturer, is not guaranteed or endorsed by the publisher.

## Footnotes

^1^ https://www.psychometrica.de/effect_size.html
